# Update on Intra-Arterial Chemotherapy for Retinoblastoma

**DOI:** 10.1155/2014/869604

**Published:** 2014-10-22

**Authors:** Mario Zanaty, Guilherme Barros, Nohra Chalouhi, Robert M. Starke, Philip Manasseh, Stavropoula I. Tjoumakaris, Carol L. Shields, David Hasan, Ketan Bulsara, Robert H. Rosenwasser, Pascal Jabbour

**Affiliations:** ^1^Department of Neurosurgery, Thomas Jefferson University and Jefferson Hospital for Neuroscience, Philadelphia, PA 19107, USA; ^2^Department of Neurosurgery, University of Virginia, Charlottesville, VA 22908, USA; ^3^Department of Ophthalmology, Thomas Jefferson University Hospital, Philadelphia, PA 19017, USA; ^4^Department of Neurosurgery, University of Iowa School of Medicine, Iowa City, IA 52242, USA; ^5^Department of Neurosurgery, Yale University School of Medicine, New Haven, CT 06510, USA; ^6^Department of Neurological Surgery, Division of Neurovascular Surgery and Endovascular Neurosurgery, Thomas Jefferson University Hospital, 909 Walnut Street, 2nd Floor, Philadelphia, PA 19107, USA

## Abstract

The tools for managing retinoblastoma have been increasing in the past decade. While globe-salvage still relies heavily on intravenous chemotherapy, tumors in advanced stage that failed chemotherapy are now referred for intra-arterial chemotherapy (IAC) to avoid enucleation. However, IAC still has many obstacles to overcome. We present an update on the indications, complications, limitations, success, and technical aspects of IAC. Given its safety and high efficacy, it is expected that IAC will replace conventional strategies and will become a first-line option even for tumors that are amenable for other strategies.

## 1. Introduction

Retinoblastoma is the most common intraocular malignancy in children [1], leading to death within 1-2 years if left untreated [1]. Survival after diagnosis mirrors economic development and ranges from 30% in Africa to 60% in Asia and 95–97% in Europe and North America [1]. Over the last few years, intra-arterial chemotherapy (IAC) has surfaced as a promising treatment alternative for advanced and refractory retinoblastomas. The main reason behind the increased popularity of IAC is to avoid enucleation. Today, IAC is increasingly gaining ground as an effective and safe treatment in the management of retinoblastoma [2, 3]. However, IAC still has many obstacles to overcome. We present an update on IAC for retinoblastoma apropos the techniques, limitations, and outcomes.

## 2. Tools for Management

Establishing an accurate diagnosis and staging the disease are the first steps in the management of retinoblastoma to avoid mistreating the patient with chemotherapy. The physician cannot always easily distinguish between retinoblastoma and mimicking lesions, called pseudoretinoblastomas. In a review of 2775 patients referred for management for retinoblastoma, 78% were confirmed with retinoblastoma while 22% had pseudoretinoblastomas [4]. Pseudoretinoblastoma comprises a range of diagnosis, such as persistent fetal vasculature, Coats disease, vitreous hemorrhage, toxocariasis, and familial exudative vitreoretinopathy [4]. The prevalence of these mimickers depends mostly on the child's age [4]. The tools for management of retinoblastoma include enucleation, radiotherapy (teletherapy or brachytherapy), chemotherapy, cryotherapy, laser photocoagulation, and transpupillary thermotherapy [5]. However, globe salvage still relies heavily on chemotherapy [6].

Chemotherapy can use various routes, such as intravenous, periocular, intravitreal, or intra-arterial. The chemotherapy strategy depends mostly on the severity of the ocular tumor, for which the most commonly used classification is the International Classification of Retinoblastoma [7] (Table 1). This presurgical classification is favored by many authors as it has been found predictive of treatment success following intravenous chemotherapy (IVC) [6]. Ocular tumors are separated into five letter groups from A to E of increasing disease progression. The grouping is based on specific ophthalmoscopic features, such as the presence of vitreous or subretinal seeding. Each group has a corresponding risk of treatment failure and subsequent enucleation, with the lowest risk in group A and the highest risk in group E [7].

## 3. Update on the General Treatment Strategy

IVC has been effective for the control of intraocular disease, prevention of metastasis, and reduction in the prevalence of pinealoblastoma and long-term second malignant neoplasms [8]. It has the additional benefits of minimal systemic toxicity and no adverse ocular effects [8]. Therefore, IVC is recommended as the first line treatment for germ-line mutation retinoblastomas (bilateral, familial), particularly to protect patients from pineoblastoma and second cancers and, in addition, to achieve control of the intraocular retinoblastoma and to prevent metastasis [6].

On the other hand, non-germ-line mutation retinoblastomas (unilateral) are best managed with IAC, if the disease is judged to be beyond control by local treatment modalities such as laser photocoagulation, thermotherapy/cryotherapy, or plaque radiotherapy. IAC is also indicated for unilateral advanced disease. Unilateral retinoblastoma of groups D and E was classically managed with enucleation, but now it can be managed with globe-conserving strategies, that is, IVC combined with IAC [8]. Advanced cases may also benefit from periocular chemotherapy combined with IVC.

Finally, intravitreal chemotherapy can be used as a last resort, following incomplete control with IAC [8], specifically when recurrent vitreous seeds are present. In fact, the main reason for IAC failure and subsequent enucleation is the recurrence of subretinal or vitreous seeds. We might be able to overcome this problem of recurrent subretinal/vitreous seeds with the use of intravitreal melphalan, with or without topotecan. Reports of higher rates of global salvage using multimodality treatments are already available [9, 10]. Bilateral groups D and E retinoblastoma cases should receive an additional subtenon carboplatin boost for improved local control [8]. The most common causes for undergoing enucleation are tumor size, poor visual improvement potential, and risk of metastatic disease [8].

## 4. Update on Technical Aspects

The IAC technique usually relies on navigating through the ipsilateral internal carotid artery to selectively catheterize the ostium of the ophthalmic artery (OA) (with a Prowler-10 microcatheter, Cordis Neurovascular). This is followed by a superselective injection to confirm the position of the microcatheter and to ascertain the lack of reflux into the internal carotid artery [11]. If a direct catheterization of OA is not successful, two alternatives routes can be safely and effectively used [12]. The first is the catheterization of the OA through the middle meningeal artery (MMA), if a communicating branch between the 2 systems is well developed [11]. If an anastomosis is not sufficiently developed, the “Japanese technique” can be used. This technique involves rapid chemotherapy delivery through a catheter placed in the internal carotid artery, at the takeoff of the OA, with balloon occlusion of distal flow [13].

At present, the superiority of direct catheterization over the Japanese technique is uncertain. In theory, direct catheterization minimizes the systemic absorption and maximizes the drug availability at the tumor bed. Even though this approach is technically difficult, the rate of technical success in expert hands is as high as 98.5% to 100% [11], supporting the evidence of a learning curve. Limitations include the risk of endothelial injury [14] and OA thrombosis, necessitating the use of alternative routes (described above). The Japanese technique or balloon-assisted catheterization also carries a technical challenge and is limited by the risk of ischemic and thromboembolic events.

In a retrospective review, Klufas and colleagues [12] reported tumor control in 17 out of 18 eyes, using IAC via the alternative routes, at a mean followup of 18.9 months. The treatment routes included catheterization via the MMA, balloon-assisted infusion alone, and balloon-assisted infusion in conjunction with direct OA catheterization. Sometimes a combination of two or all three modalities was necessary. The authors concluded that when direct catheterization is not possible, the alternative techniques can be used safely to preserve vision with acceptable side effects [12].

## 5. Outcomes of IAC: Update on Success and Advantages

IAC offers the advantage of decreasing the systemic distribution of the given drug, consequently minimizing drug-related toxicities including neutropenia, anemia, and secondary neoplasms [15]. Reducing the systemic absorption allows for the use of highly potent drugs, namely, melphalan [11], which is proven to be the most effective chemotherapeutic agent against retinoblastoma. Melphalan is very toxic at therapeutic levels when used systemically [16], but it can be safely used via the intra-arterial route [11]. Undeniably, melphalan remains as the ideal agent for IAC due to its efficacy and short half-life [17, 18]. Melphalan is often used alone but can be combined with topotecan for advanced cases with extensive vitreous seeds [17, 19]. Another advantage of minimizing the systemic toxicity is the decreased need for hospitalization, allowing the child to be discharged the same day, in the absence of intraoperative vascular complications. Furthermore, IAC allows the administration of significantly higher doses of chemotherapy directly to the tumor bed. This enhances the biological effect, improves the tumor control, and, thus, reduces the rate of recurrence [15].

For all the reasons discussed above, IAC has emerged as an appealing approach in the management of retinoblastoma. However, it is important that these advantages offer increased survival and better quality of life. So far, IAC has achieved impressive results in the treatment of refractory retinoblastomas, shrinking tumor size, and decreasing the rate of enucleation [3, 17, 18, 20, 21]. The superiority of IAC lies in its unparalleled ability to cure resistant tumors, using only 1 chemotherapeutic agent most of the time. Gobin et al. [17] reported successful catheterization in 98% of procedures with an ocular survival rate at 2 years of 82%, when IAC was the primary treatment, and 58%, when it was secondary treatment (after external beam radiation or systemic chemotherapy). Peterson et al. [21] treated 17 eyes, 16 of which had already failed other modalities, with intra-arterial melphalan, dramatically reducing the rate of enucleation from 100% to 23.5%. Shields et al. [18] found primary therapy IAC to successfully achieve globe salvage in 100% of group C, 100% of group D, and 33% of group E eyes. In the same report [18], globe salvage was successfully achieved in 50% of cases when IAC was a secondary treatment.

In another retrospective review of 70 eyes (60 patients), IAC as a primary therapy achieved globe salvage in 100% of group B, 100% of group C, 94% of group D, and 36% of group E [6]. Of all 70 eyes, complete regression was achieved for solid tumors in 48 of 51 eyes (94%), subretinal seeds in 40 of 42 eyes (95%), and vitreous seeds in 34 of 39 eyes (87%) [6]. After a mean followup of 19 months, authors reported globe salvage in 72% of primary-treated cases and 62% of secondary-treated cases. Failure and subsequent enucleation were due to tumor recurrence in only one case of primary therapy IAC, whereas the other cases of failure were due to recurrent subretinal or vitreous seeding, across all groups, with the majority in advanced group E. Furthermore, after 2 years of followup, the authors did not find any evidence of retinoblastoma metastasis or of a second cancer. No deaths or life-threatening complications have been reported.

**Figure 1:**
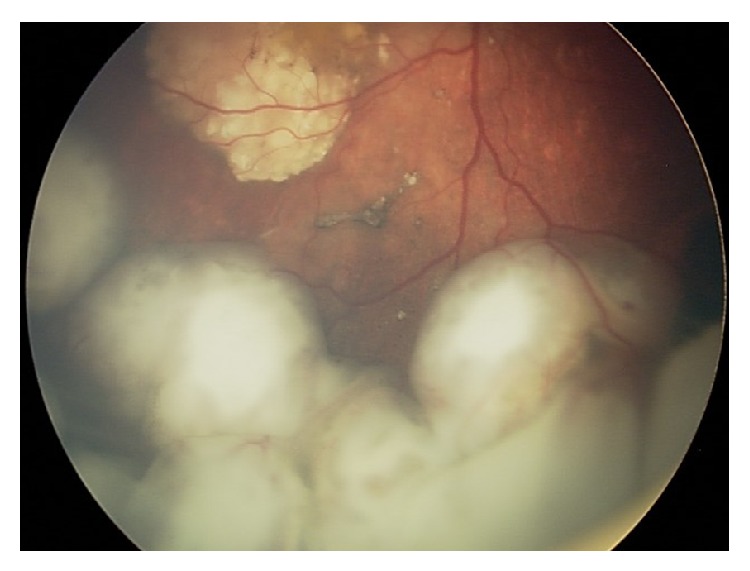
Fundoscopic exam revealing an intraocular retinoblastoma before treatment with intra-arterial chemotherapy.

**Figure 2:**
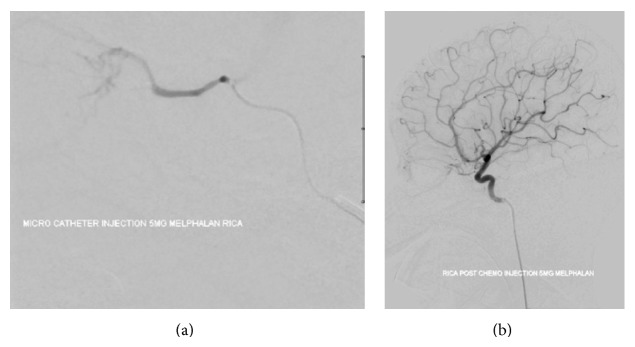
Digital subtraction angiography showing superselective catheterization with melphalan injection of the ophthalmic artery.

**Figure 3:**
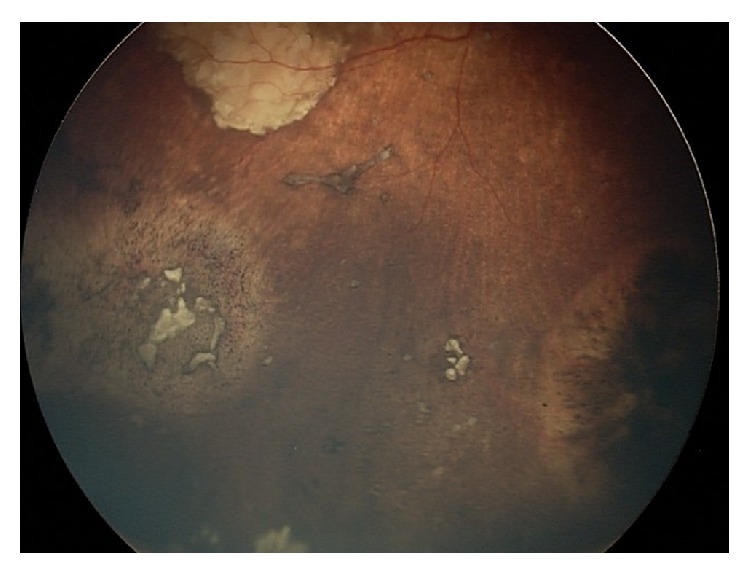
Fundoscopic exam revealing the same patient with intraocular retinoblastoma after treatment with intra-arterial chemotherapy.

A suitable option to avoid enucleation after IAC failure would be the use of intravitreal melphalan, with or without topotecan, for extensive vitreous seeding. This technique has been reported to achieve up to 100% control of vitreous seeding [9, 10, 22]. It is also reported that combined intravitreal chemotherapy and IAC for advanced group D and E eyes can lead to globe salvage in 57% of the cases [23]. This is extremely important, as these patients often have bilateral disease and would otherwise be destined for systemic chemotherapy with enucleation. Finally, IAC was shown to be effective in retinoblastomas with retinal detachment. Partial retinal detachment showed complete resolution, and complete reattachment was noted in most cases of full retinal detachment [23]. We present one illustrative case (Figures 1, 2, and 3).

## 6. Updates on Limitations

### 6.1. General Limitations

IAC has some limitations. It is less effective for advanced group E eyes (as reported above) and for tumors with vitreous seeding (higher recurrence rate). It was also theorized that the absence of systemic absorption, while being advantageous for reducing toxicity, leads to inadequate elimination of micrometastasis and extraocular tumor cells [15]. Without enucleation, tumor invasion and histopathologic features suggestive of metastasis cannot be assessed. Therefore, the patient would not receive the indicated adjuvant systemic chemotherapy that he or she would have otherwise received if enucleation had occurred. Likewise, when compared with systemic chemotherapy, IAC might not provide sufficient protection against pineoblastoma and secondary tumors, due to the lack of adequate systemic absorption of the drug [11]. This risk, however, is mostly present in children with germ-line mutations. Of all the limitations to date, the most concerning issue that IAC faces to date is the risk of metastatic disease.

### 6.2. Economical Burden

Despite the comparable cost of systemic chemotherapy and IAC per episode, the current strategy of multiple planned IAC sessions makes it significantly more costly [24]. In fact, a study published in 2012 reported that the lowest-cost treatment strategy per episode of care is enucleation ($48,000), followed by focal laser therapy ($100,250), systemic chemotherapy alone ($253,000), systemic chemotherapy with planned enucleation ($281,000), and lastly IAC with melphalan ($160,000 for 3 cycles, $310,000 for 6 cycles) [24]. IAC can cost up to $430,000 for bilateral cases. These costs reflect the hospital charges per episode of care and do not include the costs of follow-up visits, complications, and nonmedical indirect costs [24]. Although enucleation is more cost effective, it should be used as a last resort, given the risk of potential contralateral disease and the problem of leaving the child with monocular vision. Enucleation decreases visual acuity (lack of binocular summation) and the visual field, impairs space orientation and depth perception, and has psychological effects on the child. While enucleation is less favored in developed countries, where globe-salvaging options are readily available, it is still widely used in developing countries, where children have no access to sophisticated tertiary centers.

### 6.3. Complications

Complications following IAC can result from the technique itself, the chemotherapeutic agents, or both (Table 2). The complications attributed to the technique are iodine allergies, complications at the femoral puncture site, and intraoperative endovascular complications. In the current literature, thromboembolic and hemorrhagic strokes have rarely occurred [11] with carotid vascular spasm, stroke, and MRI displaying focal perfusion defects [8]. Transient pancytopenia from bone marrow suppression may occur, but neutropenia and anemia rarely require intervention. Local, minor ocular toxicities include eyelid edema, forehead erythema, thinning or loss of eyelashes, blepharoptosis, and transient ocular dysmotility [17, 18], which are all usually transient. Major ocular toxicities are mostly the result of vascular complications involving the retinal, ophthalmic, and choroidal arteries.

Munier et al. [25] reported that 23% (3/13) of patients had choroidal ischemia or retinal arteriolar embolism. Shields et al. [18] reported choroidal vasculopathy or retinal artery occlusion in 35% (6/17) of cases. Muen et al. [28] reported ocular side effects of cranial nerve palsy (40%), orbit/eyelid edema (20%), retinal detachment (7%), vitreous hemorrhage (27%), and retinal pigment epithelial changes (47%). The retinal pigment epithelial changes could be related to previous retinal detachment or choroidal vascular compromise [8]. In a more recent study [6], the main complications following IAC, per catheterization, included transient eyelid edema (5%), blepharoptosis (5%), forehead hyperemia (2%), vitreous hemorrhage (2%), branch retinal artery obstruction (1%), ophthalmic artery spasm with reperfusion (2%), ophthalmic artery obstruction (2%), partial choroidal ischemia (2%), and optic neuropathy (<1%). There were no patients with stroke, seizure, neurologic impairment, limb ischemia, secondary leukemia, metastasis, or death [6]. Furthermore, the authors report that the combined incidence of ophthalmic, retinal, and choroidal vascular ischemia has reduced to 1% [6], again pointing toward the presence of a learning curve.

Management of a child with retinoblastoma involves a balance of patient life with globe salvage and ultimate visual potential [26, 29]. While intravenous chemotherapy is known to have no local toxic effects on the eye related to vision [30], the visual outcome after IAC is not well evaluated. The relevant question is whether sparing the globe and using IAC translate to visual success. Munier et al. [25] found 31% (4/13) of patients receiving IAC retained vision of 20/50 or better but did not state the proportion of patients with foveae involvement before treatment, which is an independent predictor of poor vision [30]. Tsimpida and colleagues examined the visual outcome in patients with refractory retinoblastoma who had previously undergone systemic chemotherapy, with or without local treatment, and were subsequently treated with IAC [27]. Five of twelve eyes (42%) demonstrated severe visual loss at last followup. The reported causes were retinal detachment (1 eye, 20%) and choroidal ischemia involving the foveae (4 eyes, 80%). All 3 patients who had difficulties or vasospasm during catheterization and all patients who received the nonage-adjusted dose of melphalan suffered from visual loss, while those who received the age-adjusted dose did not. The authors suggested any form of radiation before IAC is associated with worse complications, and despite the fact that the exact mechanism of vision deterioration after IAC is unknown, melphalan use and difficulty catheterizing the OA are compelling factors. Thus, based on these findings, an optimal melphalan dose needs to be determined, where maximal tumor control and preservation of visual function are achieved [27]. One final problem that remains with IAC is the repetitive exposure to ionizing radiation, which could potentially harm the thyroid, bone marrow, and other susceptible organs. This is one of the areas where advances are needed to avoid excess exposure to radiation in our young patients. However, it is still not clear whether repetitive exposure to low dose radiation has an additive effect or not.

## 7. Conclusion

IAC has emerged as a remarkably effective strategy for treating retinoblastoma. IAC is effective as both primary and secondary treatment. Given its reported safety and high efficacy, it is expected that IAC will replace conventional strategies and will become a first-line option, even for tumors amenable to other strategies.

As with the introduction of any novel treatment, studies show that technical complications are decreasing with growing experience, supporting the existence of a learning curve. IAC has allowed eyes that would have been enucleated in the past to be salvaged, with the vast majority of treated patients today retaining their eyes. In the future, even more globes will be salvaged, since the combination of IAC with intravitreal chemotherapy and other modalities is achieving higher success and superior tumor control.

## Figures and Tables

**Table 1:** The International Classification of Retinoblastoma (IRCB) [7].

Group	Defining features
A	Tumor <3 mm in size
B	Tumor >3 mm in size or macular Rb location; juxtapapillary Rb location, clear subretinal fluid ≤3 mm from margin
C	Tumor with focal seeding: subretinal seeds ≤3 mm from Rb; vitreous seeds ≤3 mm from Rb; subretinal and vitreous seeds ≤3 mm from Rb
D	Tumor with diffuse seeding: subretinal seeds >3 mm from Rb; vitreous seeds >3 mm from Rb; subretinal and vitreous seeds >3 mm from Rb
E	Extensive tumor occupying >50% of the globe or neovascular glaucoma; invasion of postlaminar optic nerve, choroid, sclera, orbit, anterior chamber; massive intraocular hemorrhage

^∗^Rb: retinoblastoma.

**Table 2:** Complications of intra-arterial chemotherapy for retinoblastoma in the literature.

Studies	Number of patients	Choroidal ischemia (%)	Retinal detachment	Eyelid edema	Other
Munier et al. [25]	13	23%			

Shields et al. [18]	17	35%			

Bayar et al. [26]	15		7%	20%	(i) CN palsy 7% (ii) Vitreous hemorrhage 27% (iii) Retinal pigment epithelial change 47%

Shields et al. [6]	70			14%	(i) Blepharoptosis 14% (ii) Forehead hyperemia 4% (iii) Vitreous hemorrhage 6% (iv) Ophthalmic artery obstruction 4%

Tsimpida et al. [27]	12	33%	8%		Total visual loss 4%

Gobin et al. [17]	98			14%	
